# Supplementary material for: CSN5A Subunit of COP9 Signalosome Is Required for Resetting Transcriptional Stress Memory after Recurrent Heat Stress in Arabidopsis
Source: Biomolecules. 2021 Apr 30;11(5):668. doi: 10.3390/biom11050668 (PMC8146153; doi:10.3390/biom11050668)
Supplement: Supplementary file 1 [file biomolecules-11-00668-s001.zip › biomolecules-1185968-supplementary.pdf]

Table S1: Primers used in this research work

Gene	Primer sequence 5'- 3' for qRT-PCR
APX2 F	CTTGATGATCCTCTCTTTCTCCCA
APX2 R	ACTCCTTGTCAGCAAACCCGAG
HSP22 F	ATTCTGGAGACAGTTCAAGCTACCT
HSP22 R	TTCAGGAGATAGTTTCGTGAGGTTA
HSP70 F	CCGTCTTCGATGCTAAGCGTCT
HSP70 R	AACCACAATCATAGGCTTCTCACC
ACTIN 8 F	CACTTTCCAGCAGATGTGGATC
ACTIN 8 R	AATGCCTGGACCTGCTTCAT

	Primers sequence 5'-3' for Histone ChIP	Amplicon region
APX2 F	GGATATCAAACCCAACTTGAAGAGAG	1
APX2 R	ATAATCTGAGCAAAAGATAAAACACGG	1
APX2 F	CTGTTCCCTATTCTGTCATATGCTG	2
APX2 R	ACCCTTGATTCTATGGTTCTACCTC	2
HSP22 F	CGTTGGACTTGGCCTTAGAT	1
HSP22 R	TGACTGCTCCCTGATTCTTG	1
HSP22 F	ATGAAGCACTTGCTAAGCATCTTC	2
HSP22 R	AGTCCTAATGGGATTCTCTCCA	2
HSP70 F	TCAAAAGGGGTCTACGGAAA	1
HSP70 R	GCACCACACGTTTTCAAGGT	1
HSP70 F	CCACTCTTCATTCATATATAAACA	2
HSP70 R	ATTCGTCTGTGAGCTTTAAGAG	2
